# Preoperative prediction of cytokeratin-19 expression for hepatocellular carcinoma using T1 mapping on gadoxetic acid-enhanced MRI combined with diffusion-weighted imaging and clinical indicators

**DOI:** 10.3389/fonc.2022.1068231

**Published:** 2023-01-19

**Authors:** Yue Zhao, Xiaoliang Tan, Jingmu Chen, Hongweng Tan, Huasheng Huang, Peng Luo, Yongsheng Liang, Xinqing Jiang

**Affiliations:** ^1^ Department of Radiology, The First Affiliated Hospital of Jinan University, Guangzhou, China; ^2^ Department of Radiology, Guangzhou First People’s Hospital, Guangzhou, China; ^3^ Department of Radiology, Central People's Hospital of Zhanjiang, Zhanjiang, China

**Keywords:** hepatocellular carcinoma, cytokeratin 19, magnetic resonance imaging, T1 mapping, nomogram

## Abstract

**Objectives:**

To explore the value of T1 mapping on gadoxetic acid-enhanced magnetic resonance imaging (MRI) in preoperative predicting cytokeratin 19 (CK19) expression for hepatocellular carcinoma (HCC).

**Methods:**

This retrospective study included 158 patients from two institutions with surgically resected treatment-native solitary HCC who underwent preoperative T1 mapping on gadoxetic acid-enhanced MRI. Patients from institution I (n = 102) and institution II (n = 56) were assigned to training and test sets, respectively. univariable and multivariable logistic regression analyses were performed to investigate the association of clinicoradiological variables with CK19. The receiver operating characteristic (ROC) curve and precision-recall (PR) curve were used to evaluate the performance for CK19 prediction. Then, a prediction nomogram was developed for CK19 expression. The performance of the prediction nomogram was evaluated by its discrimination, calibration, and clinical utility.

**Results:**

Multivariable logistic regression analysis showed that AFP>400ng/ml (OR=4.607, 95%CI: 1.098-19.326; *p*=0.037), relative apparent diffusion coefficient (rADC)≤0.71 (OR=3.450, 95%CI: 1.126-10.567; *p*=0.030), T1 relaxation time in the 20-minute hepatobiliary phase (T1rt-HBP)>797msec (OR=4.509, 95%CI: 1.301-15.626; *p*=0.018) were significant independent predictors of CK19 expression. The clinical-quantitative model (CQ-Model) constructed based on these significant variables had the best predictive performance with an area under the ROC curve of 0.844, an area under the PR curve of 0.785 and an F1 score of 0.778. The nomogram constructed based on CQ-Model demonstrated satisfactory performance with C index of 0.844 (95%CI: 0.759-0.908) and 0.818 (95%CI: 0.693-0.902) in the training and test sets, respectively.

**Conclusions:**

T1 mapping on gadoxetic acid-enhanced MRI has good predictive efficacy for preoperative prediction of CK19 expression in HCC, which can promote the individualized risk stratification and further treatment decision of HCC patients.

## 1 Introduction

Hepatocellular carcinoma (HCC) is the third most common cause of cancer-related death worldwide, with an increasing incidence and mortality in the past 20 years (1). Although the prognosis of patients with HCC has improved with advances in surgical and imaging technology, the high rates of intrahepatic recurrence after hepatectomy still remain a major challenge for treatment of HCC, and two thirds of recurrences are within 5 years (2, 3). Recurrence after hepatectomy may be associated with the degree of differentiation, microvascular invasion, satellite focus and related gene expression, among which cytokeratin 19 (CK19) expression is considered to be an important influencing factor (4).

CK19 is well acknowledged as a biliary/progenitor cell marker and a tumor stem cell marker, and plays an important role in promoting malignant property of HCC (5). Compared with patients with CK19-negative HCCs, CK19-positive HCCs is associated with clinical aggressiveness due to more tumor invasion, higher rate of lymph node metastasis intrahepatic recurrence, and poorer prognosis after resection and liver transplantation (6, 7). Therefore, understanding CK19 expression status of HCC at diagnosis can be significant for better clinical decision making and improved prognosis. However, since CK19 expression status can only be diagnosed histologically (8), its use as a prognostic indicator for treatment allocation is limited.

Several studies have assessed the imaging findings associated with poor prognosis for CK19-positive HCCs. For instance, irregular tumor margin, rim arterial phase hyperenhancement, lower tumor-to-liver apparent diffusion coefficient (ADC) ratio, and lower tumor-to-liver signal intensity (SI) ratio at hepatobiliary phase (HBP) imaging are considered to be significant independent variables for potentially predicting CK19-positive HCCs (9–11). Radiomics can extract a large number of high-dimensional quantitative features from multimodal medical images, and then reveal the correlation between these features and the diagnosis, pathology, and prognosis of the tumor (12). Recently, studies have used radiomics based on MRI to predict CK19 in HCCs (13–16). Radiomics has progressed quite significantly, but the following problems remain. First, accurate image segmentation relies on manual delineation, which is time-consuming and easily affected by the operator. Second, the different designs of the image features can lead to different analytic results (17, 18). Unlike traditional radiomics, Deep learning (DL) models are capable of automatic learning, extracting, and selecting image features for prediction, and thereby can more comprehensively and profoundly excavate information from images. Chen Y et al. (19) fully utilized the image information including intratumoral and peritumoral regions of the hepatic lesions through a DL algorithm, providing more valuable relevant information for better prediction of CK19 expression. Although DL-based model is a promising approach, there are still some problems in the application, such as the black box problem, non-transparency of decision-making, and difficulty in interpretation from the clinical aspect (20). Thus, a feasible and quantitative method is urgently needed to predict CK19 expression in HCC patients. T1 mapping is a non-invasive method for quantitative analysis of T1 value in tissues. It reflects the intrinsic characteristics of tissues and is not affected by scanning sequence parameters (21). Moreover, it is positively proportional to the concentration of gadolinium contrast agent in tissues, and can more accurately and objectively reflect the uptake of gadoxetic acid (22). To our knowledge, the quantitative evaluation of CK19 expression of HCC using T1 mapping has not been reported. Therefore, the purpose of this study was to preoperatively identify CK19 expression status of HCC by T1 mapping on gadoxetic acid-enhanced MRI.

## 2 Materials and methods

### 2.1 Study patients

**Figure 1:**
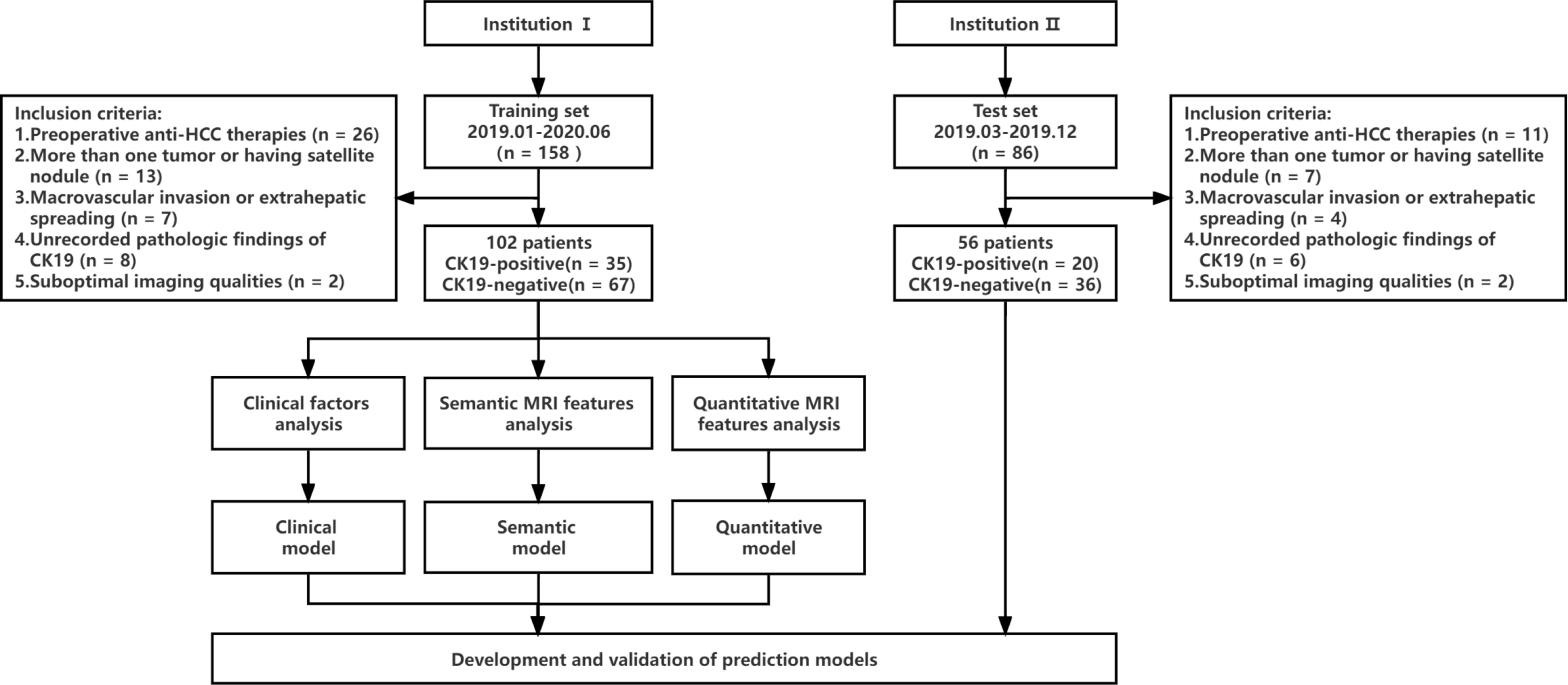
Study flowchart. CK19, Cytokeratin 19; HCC, hepatocellular carcinoma.

This study was a retrospective study, which was approved by the hospital ethics Committee, and patients were exempted from signing informed consent. The flow chart of data collection and research design is shown in **Figure 1**. We retrospectively collected patients from Zhanjiang Central People’s Hospital (Institution I) and Guangzhou First People’s Hospital (Institution II). Inclusion criteria were as follows (1): Single HCC was pathologically diagnosed after hepatectomy and immunohistochemical examination of CK19 was performed (2); Gadoxetic acid-enhanced MRI examination was performed within 2 weeks before surgery, including T1 mapping in the pre-enhanced and 20-minute hepatobiliary phase (HBP) after gadoxetic acid injection (3); Complete clinical and pathologic data. Exclusion criteria were as follows (1): administration of other preoperative antitumor therapies, such as radiofrequency ablation, transcatheter arterial chemoembolization (TACE), etc. (2); more than one tumor or having satellite nodules (3); presence of macrovascular invasion or extrahepatic spreading (4); unrecorded pathologic findings of CK19 (5); suboptimal MR image quality for interpretation. MR images of 102 patients from institution I were used as the training set to establish prediction models to predict CK19 expression in HCC. The predictive performances of models were evaluated by test sets (56 cases from institution II).

### 2.2 Clinicopathological analyses

Preoperative laboratory indicators included Alpha Fetoprotein (AFP), Alanine aminotransferase (ALT), Aspartate aminotransferase (AST), Glutamyl transpeptidase (GGT), Alkaline phosphatase (ALP), Albumin (ALB), Total bilirubin (TBIL), Direct bilirubin (DBIL), Serum creatinine (Scr), Prothrombin time (PT) and International Normalized ratio (INR), Neutrophils to lymphocyte ratio (NLR), Platelet to lymphocyte ratio (PLR).

The diagnostic criteria for HCC were based on morphological criteria defined by the World Health Organization. The expression of CK19 was semiquantitatively evaluated by immunochemical staining. The hepatocytes and bile ducts of normal liver tissues were used as negative and positive controls, respectively. Tumors were classified as negative (<5% of tumor cells) or positive (≥5% of tumor cells) for CK19 by an experienced pathologist who was blinded to clinical and imaging information.

### 2.3 Magnetic resonance imaging protocol

MRI examinations in all patients from institution I and institution II were performed using a 1.5T (Magnetom Aera; Siemens Healthcare) and 3.0T (Magnetom Trio A Tim; Siemens Healthcare) MR scanner, respectively. The scanning range covered from the top to the lower edge of the liver with an 8-channel phased-array coil as the receiver coil. Gadoxetic acid-enhanced MRI was obtained including the pre-enhanced, enhanced arterial phase (AP, 20–40s), portal phase (PVP, 50–70s), equilibrium phase (EP, 100–120s), and 20min HBP images. Gadoxetic acid (Primovist; Bayer Schering Pharma, Berlin, Germany) was injected into the cubital vein at a flow rate of 1.0 ml/s and a dose of 0.025 mmol/kg, followed by 20 ml of normal saline for flushing. A more detailed description of the MRI methods and specific sequences and parameters of MRI scans are shown in **Supplementary Materials 1.1** and **Table S1**.

### 2.4 Imaging analysis

Preoperative MRI images were retrospectively analyzed on the Picture Archiving and Communication System (PACS). The semantic and quantitative MRI features were evaluated by two abdominal radiologists independently (reader 1 [TAN XL] and reader 2 [CHEN JM] both with 6 years of experience in liver imaging) who were blinded to the patients’ clinical and pathologic information. Discrepancies in semantic features were resolved by consensus after reevaluating the images. Quantitative characteristics were obtained by averaging the estimates of two readers. One reader [TAN XL] repeated the assessment in the same manner after 2 weeks to minimize the memory effect to evaluate intraobserver agreement. The intra- and inter-class correlation coefficients (ICCs) were calculated to measure the intra- and inter-rater reproducibility of semantic and quantitative characteristics, respectively.

Semantic MRI features include 1) Tumor margin; 2) Hemorrhage; 3) Necrosis; 4) Fat component; 5) Target sign; 6) Washout; 7) Rim Arterial phase hyperenhancement (rim APHE); 8) Corona enhancement; 9) Intratumoralarteries; 10) Radiologic capsule; 11) Peritumoral hypointensity on HBP. A detailed description of semantic MRI features is provided in **Supplementary Material 1.2**. All quantitative measurements were performed manually on the PACS. The region of interest (ROI) was placed as far as possible in the area with obvious enhancement of lesion to avoid necrosis, hemorrhage, fat and artifacts. The area of ROI was about 1.0~1.5 cm^2^; the same lesion was measured three times with the same ROI, and then average amounts were calculated. The signal intensity (SI) of tumor and surrounding normal liver parenchyma were measured at Pre, AP, PVP, EP and HBP images respectively, and then the tumor to liver contrast ratio (TLR) was calculated. Additionally, ADC values of the lesion and surrounding normal liver parenchyma were measured on the ADC images, and tumor-to-liver ADC values were calculated (recorded as relative ADC, rADC); precontrast and postcontrast T1 relaxation time were measured before and 20 minutes after the administration of the contrast medium (recorded as T1rt-Pre and T1rt-HBP, respectively), and reduction rate of T1 relaxation time (rrT1rt) was calculated. The description and formulas of quantitative parameters are detailed in the **Supplemental Materials 1.2**.

### 2.5 Postoperative follow-up

All HCC patients were regularly monitored for recurrence *via* CT or MRI once every 3 months for 2 years after resection. The recurrence status included new intrahepatic lesions and/or extrahepatic metastasis and the criteria were as follows: 1) new intrahepatic lesions with typical imaging features of HCC, or confirmed by histopathology, or with tumor stain during postoperative TACE; 2) extrahepatic metastasis confirmed by typical imaging features or histopathological analysis.

### 2.6 Statistical analysis

A Student *t*-test (mean ± standard deviation) or Wilcoxon rank-sum test (median, P25 ~ P75) was performed for continuous variables. The categorical variables were compared by χ^2^. The ICCs of quantitative data between the two observers was calculated. Spearman coefficient was used for correlation analysis between quantitative parameters and CK19 status. Multivariable logistic regression analyses were performed to identify the independent predictors of CK19-positive HCCs. Akaike Information Criterion (AIC) was used to determine the optimal prediction model. The receiver operator characteristic (ROC) curve was used to evaluate the performance of predicting the expression of CK19. The comparison of different area under ROC (AUROC) curves was conducted by DeLong’s test. In view of the imbalance between the patients with CK19-negative HCCs and those with CK19-positive HCCs, we further used the F1 score and the area under the precision-recall curve (AUPRC) to compare performances, as these methods are more informative in the evaluation of binary classifiers on imbalanced data sets. Calibration curve was used to assess the consistency of nomogram. Decision Curve Analysis (DCA) was used to evaluate the clinical utility of nomogram by quantifying the net benefit under different threshold probabilities. R software (version 3.4.1) was used for analysis. All differences were considered statistically significant with a *p* value of <0.05.

## 3 Results

### 3.1 Clinicopathological features of the training and test sets

A total of 102 patients from institution I were included in this study, including 35 CK19-positive HCCs and 67 CK19-negative HCCs. 56 patients from institution II were included, among which 20 were positive and 36 were negative regarding CK19 expression. The distribution of clinicopathological features in the training and test sets is shown in **Table 1**. The results of univariable analysis of clinical factors in the training set showed that AFP and NLR were significant variables associated with CK19 expression (*P*<0.05, **Supplementary Table S2**).

**Table 1:** Baseline clinical and pathological characteristics of the training and test sets.

Characteristic	Total (n = 158)	Training set (n = 102)	Test set (n = 56)	*p* value
Age (years)	47 (42 – 63)	55 (50 - 66)	52 (45 - 63)	0.285
Gender (male)	146 (92.4%)	93 (91.2%)	53 (94.6%)	0.619
HBsAg				0.096
Negative	29 (18.4%)	18 (17.6%)	11 (19.6%)	
Positive	129 (81.6%)	84 (82.4%)	45 (80.4%)	
ALT (U/l)	34.00 (21.50 - 55.00)	34.00 (25.50 - 55.25)	31.50 (20.00 - 54.00)	0.693
AST (U/l)	41.00 (25.50 - 49.25)	42.00 (26.00 - 48.25)	41.50 (23.00 - 55.25)	0.936
GGT (U/l)	54.00 (37.50 - 118.75)	53.00 (37.50 - 109.75)	53.50 (36.75 - 120.25)	0.853
ALP (U/l)	85.50 (68.75 - 106.50)	83.00 (68.00 - 104.00)	87.00 (69.50 - 108.25)	0.457
ALB (g/l)	39.35 (36.30 - 42.42)	39.80 (36.58 - 42.75)	38.50 (35.70 - 40.70)	0.102
TBIL (umol/l)	14.62 (11.00 - 18.12)	14.82 (11.34 - 18.56)	14.30 (10.60 - 17.78)	0.685
DBIL (umol/l)	4.04 (2.70 - 6.41)	4.56 (2.81 - 6.41)	3.35 (2.40 - 5.08)	0.057
SCr (U/l)	75.50 (67.15 - 87.00)	75.00 (67.00 - 86.85)	76.00 (68.28 - 87.30)	0.648
PT (s)	11.90 (11.40 - 12.50)	11.80 (11.50 - 12.60)	11.95 (11.40 - 12.58)	0.956
INR				0.673
≤1.0	67(42.4%)	42 (41.2%)	25 (44.6%)	
>1.0	91(57.6%)	60 (58.8%)	31 (55.4%)	
NLR	2.02 (1.54 - 3.55)	2.47 (1.86 - 3.42)	2.02 (1.59 - 3.62)	0.603
PLR	106.67 (72.50 - 150.65)	109.94 (71.15 - 149.87)	105.87 (73.30 - 166.82)	0.965
AFP (ng/ml)	24.25 (4.72 - 135.41)	18.95 (4.39 - 97.42)	28.51 (5.43 - 445.00)	0.256
Edmondson-Steiner				0.394
Grade I-II	89 (56.3%)	60 (58.8%)	29 (51.8%)	
Grade III-IV	69 (43.7%)	42 (41.2%)	27 (48.2%)	
Ki-67 index	27% (10% - 40%)	20% (10% - 40%)	30% (15% - 40%)	0.369
CK-19 positive	54 (34.2%)	35 (34.31%)	19 (33.9%)	0.961

Continuous variables are presented as median (inter-quartile range, IQR). Categorial variables are presented as number (percentage). p-values represent the result of comparison of the training set with the test set.

HBsAg, hepatitis B surface antigen; ALT, alanine aminotransferase; AST, aspartate aminotransferase; GGT, glutamyl transpeptidase; ALP, alkaline phosphatase; ALB, albumin; TBIL, total bilirubin; DBIL, direct bilirubin; SCr, serum creatinine; PT, prothrombin time; INR, international normalized ratio; NLR, neutrophil to Lymphocyte ratio; PLR, platelet to Lymphocyte ratio; AFP, alpha fetoprotein; CK19, cytokeratin 19.

### 3.2 MRI features of HCCs related to CK19 expression in the training set

The results of univariable analysis of semantic features in the training set showed that CK19-positive HCCs more frequently showed nonsmooth tumor margin (*p*=0.035), target sign (*p*=0.005) and corona enhancement (*p*=0.043) compared to CK19-negative HCCs (**Supplementary Table S3**).

Agreement analysis of quantitative parameters indicated that ICCs were all above 0.70 (0.70 ~0.86, *p*<0.001, **Table 2**), which proved that the two radiologists were consistent in the analysis of quantitative features. Correlation analysis showed that T1rt-Pre (r=0.352, *p*<0.001) and T1rt-HBP (r=0.366, *p*<0.001) were moderately positively correlated with CK19. rADC (r=-0.358, *p*<0.001) and HBP-TLR (r=-0.309, *p*=0.002) were moderately negatively correlated with CK19. There was no statistical significance between other MRI quantitative parameters and CK19 (*p*>0.05) (**Supplementary Table S4**).

**Table 2:** Comparison of quantitative MRI parameters between CK19-negative and positive HCCs in training set.

	CK19-negative (n = 67)	CK19-positive (n = 35)	t/z value	*p* value	ICC	
					Intra-	Inter-
Tumor size (cm)	3.84 (2.50 - 5.75)	4.04 (2.68 - 7.66)	z=-0.219	0.223	0.86	0.83
rADC	0.88 ± 0.27	0.68 ± 0.13	t=3.838	<0.001*	0.83	0.80
AP-TLR	1.41 ± 0.38	1.31 ± 0.30	t=1.247	0.215	0.86	0.83
PVP-TLR	0.92 (0.78 - 1.24)	0.86 (0.73 - 1.05)	z=-1.589	0.112	0.82	0.79
EP-TLR	0.83 (0.72 - 1.04)	0.81 (0.68 - 0.94)	z=-1.195	0.232	0.86	0.84
HBP-TLR	0.60 ± 0.17	0.48 ± 0.18	t=3.161	0.002*	0.83	0.81
T1rt-Pre (msec)	1304.97 ± 228.39	1490.41 ± 258.21	t=-3.721	<0.001*	0.78	0.75
T1rt-HBP (msec)	757.97 ± 152.24	890.69 ± 180.92	t=-3.914	<0.001*	0.77	0.73
rrT1rt	0.41 ± 0.13	0.39 ± 0.12	t=0.571	0.570	0.73	0.70

*p<0.05. Continuous variables are presented as median (inter-quartile range, IQR) or mean ± standard deviation. Categorial variables are presented as number (percentage).

rADC, relative apparent diffusion coefficient; AP, arterial phase; PVP, portal venous phase; EP, equilibrium phase; HBP, hepatobiliary Phase; TLR, tumor to liver contrast ratio; TEI, tumor enhancement index; RTE, relative tumor enhancement; RER, relative enhancement ratio; Pre, pre-enhancement; T1rt, T1 relaxation time; rrT1rt, reduction rate of T1 relaxation time; CK-19, cytokeratin 19; ICC, intraclass correlation coefficient.

**Figure 2:**
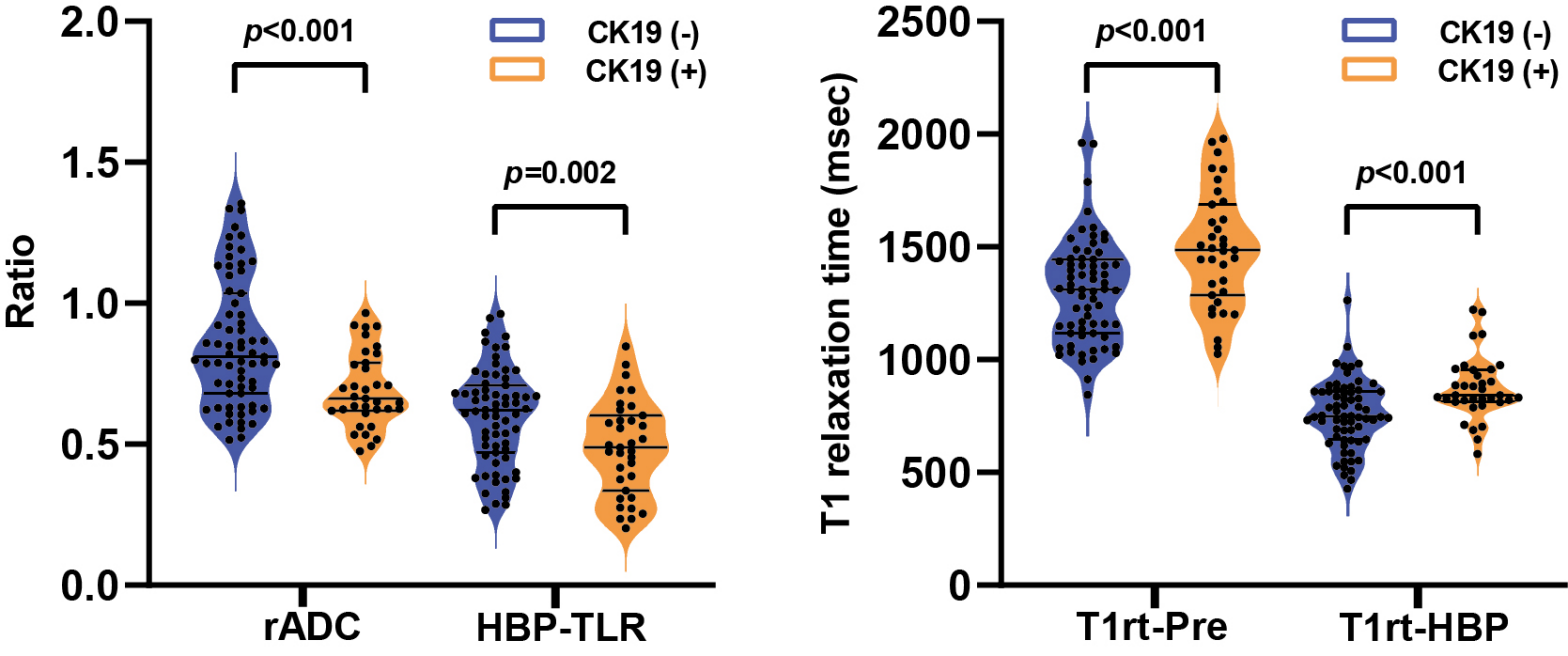
The violin plots show the comparison of relative apparent diffusion coefficient (rADC) and tumor-to-liver signal intensity ratio on the hepatobiliary phase (HBP-TLR) **(A)**, T1 relaxation time of pre-contract (T1rt-Pre) and hepatobiliary phase (T1rt-HBP) **(B)** between CK19-negative and CK19-positive groups. CK19, Cytokeratin 19; HCC, hepatocellular carcinoma.

rADC, HBP-TLR, T1rt-Pre and T1rt-HBP between CK19-positive and negative HCCs groups showed significant statistical differences (**Table 2** and **Figure 2**). The univariable discriminative performances of the above quantitative parameters were detailed in the **Supplementary Table S5**. Among these quantitative parameters, T1rt-HBP had the highest diagnostic performance in predicting CK19-positive HCCs, with an AUROC of 0.712, sensitivity and specificity of 80.00% and 62.29%, respectively. The AUC of HBP-TLR was 0.681, the sensitivity and specificity were 74.29% and 59.70%, respectively. The AUC of rADC was 0.710, the sensitivity and specificity were 68.57% and 68.66%, respectively.

### 3.3 Development and validation of predictive models for CK19-positive HCCs

Multivariable logistic regression showed AFP>400ng/ml (*p*=0.037, OR=4.607, 95%CI: 1.098-19.326), rADC ≤ 0.71 (*p*=0.030, OR=3.450, 95%CI: 1.126-10.567), T1rt-HBP>797 msec (*p*=0.018, OR=4.509, 95%CI: 1.301-15.626) were independent predictors of CK19-positive HCCs (**Supplementary Table S6**).

**Figure 3:**
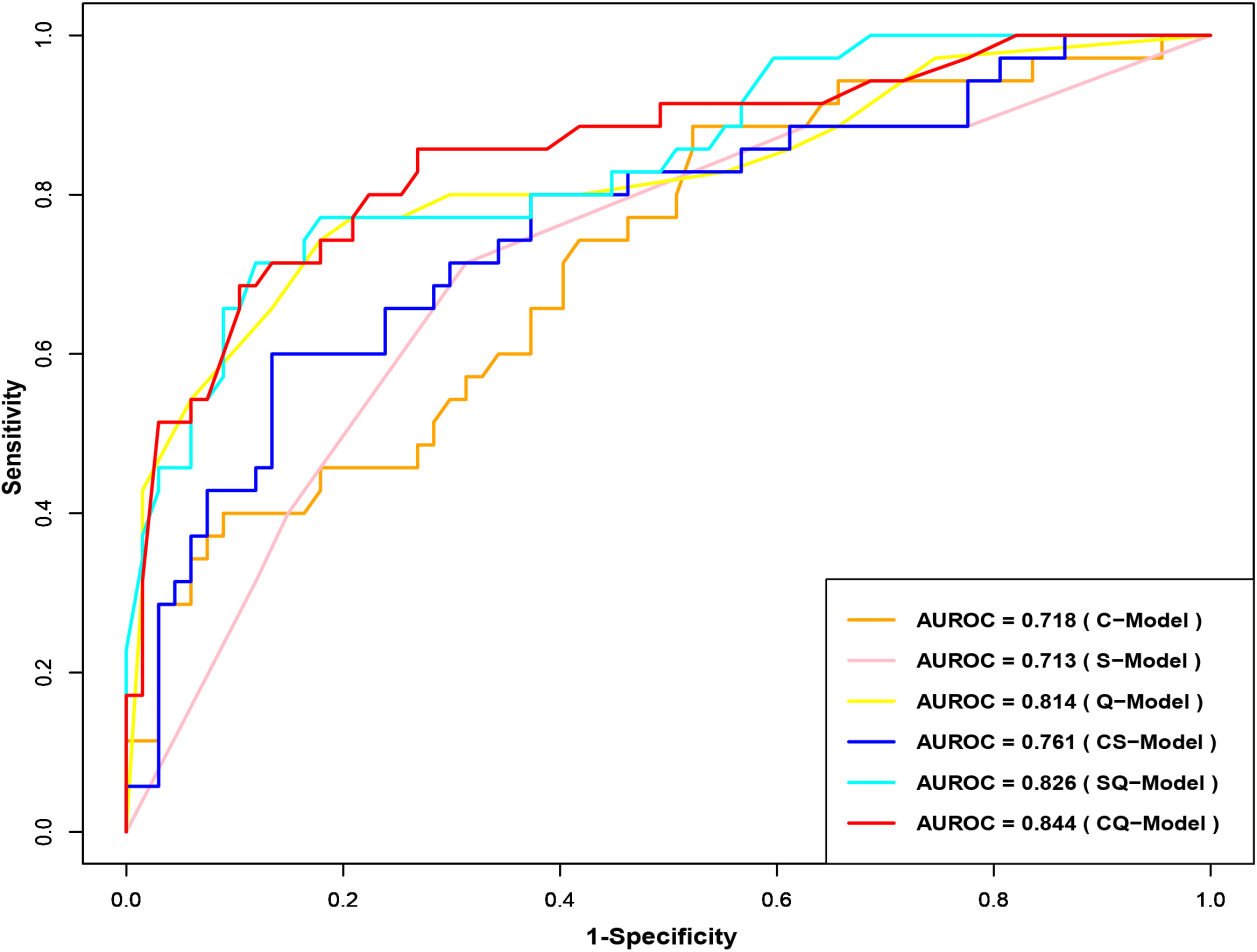
Receiver operator characteristic curves of CK19-positive HCC prediction model. CK19, Cytokeratin 19; HCC, hepatocellular carcinoma.

**Figure 4:**
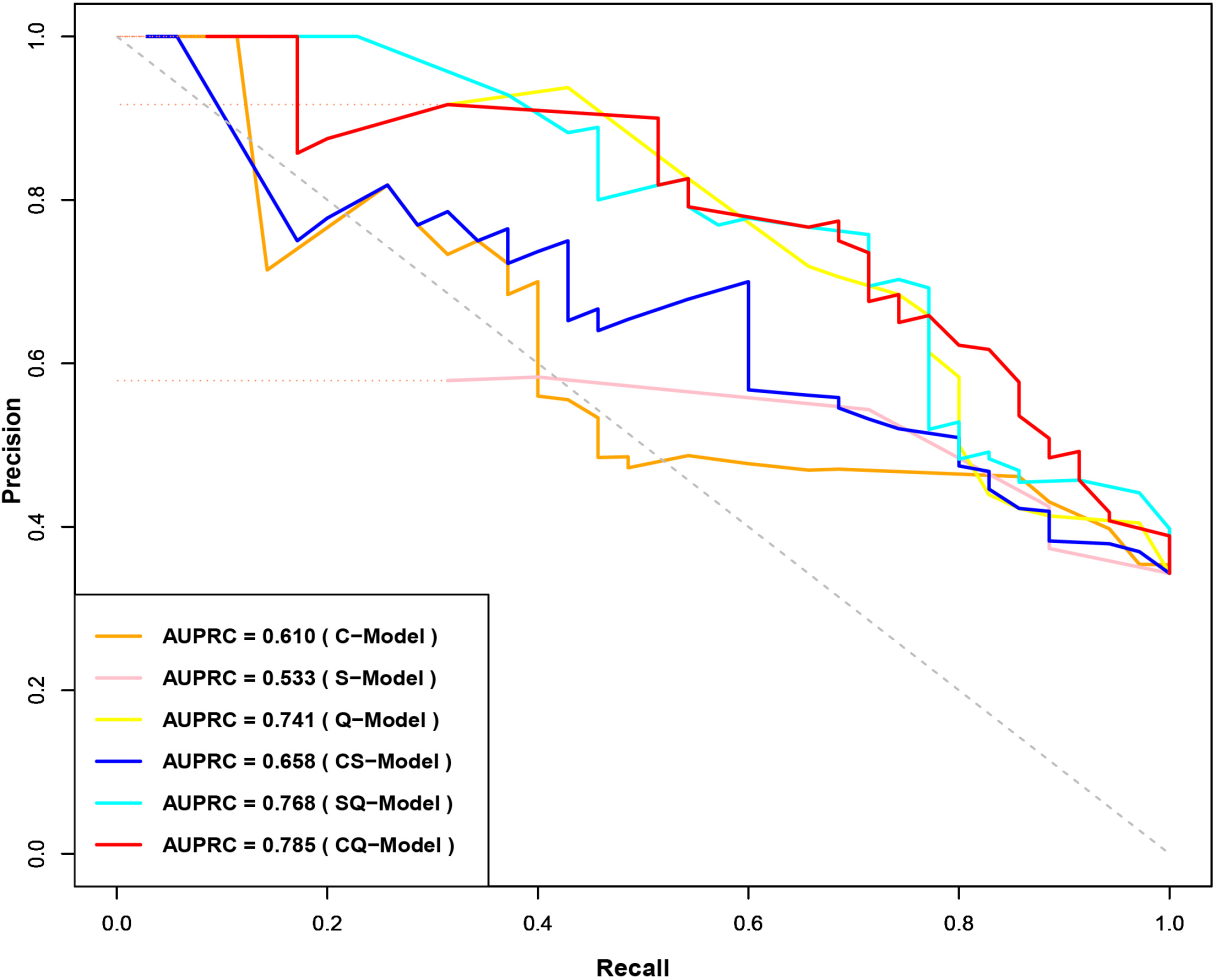
Precision-Recall Curves of CK19-positive HCC prediction model. CK19, Cytokeratin 19; HCC, hepatocellular carcinoma.

Clinical model (C-Model), semantic model (S-Model), quantitative model (Q-Model), clinical-semantic model (CS-Model), clinical-quantitative model (CQ-Model), semantic-quantitative model (SQ-Model) and clinical-semantic-quantitative model (CSQ-Model) were constructed based on clinical (AFP, NLR), semantic (nonsmooth margin, target sign, peritumoral enhancement) and quantitative (HBP-TLR, rADC, T1rt-Pre, T1rt-HBP) variables, respectively. Stepwise regressions based on the AIC were used to further select the above variables to construct multivariable logistic regression models for CK19-positive HCCs in the training dataset (**Supplementary Table S7**). Interestingly, semantic features were excluded during the stepwise regressions analysis of CSQ-Model, so the CSQ-Model was equivalent to the CQ-Model. The predictive efficacy of each model is shown in **Table 3**. CQ-model had the largest AUROC (0.844, 95%CI: 0.754-0.933) (**Figure 3**). ROC analysis among models was followed by the DeLong test to compare the predictive performance (**Supplementary Table S8**). Precision-recall curve further illustrated similar results that CQ-Model had the largest AUPRC (0.785) (**Figure 4**) and F1 score (0.778).

**Table 3:** Performance of CK19-positive HCCs prediction models in training set.

Models	ACC	SEN	SPE	PPV	NPV	AUROC	AUPRC	F1 Score
C-Model	0.617	0.886	0.477	0.469	0.889	0.718	0.610	0.613
S-Model	0.696	0.714	0.687	0.543	0.821	0.713	0.533	0.617
Q-Model	0.784	0.743	0.806	0.667	0.857	0.814	0.741	0.703
CS-Model	0.774	0.600	0.865	0.700	0.805	0.761	0.658	0.646
CQ-Model	0.843	0.800	0.866	0.757	0.892	0.844	0.785	0.778
SQ-Model	0.804	0.771	0.821	0.692	0.873	0.826	0.768	0.729

C-Model, Clinical model; S-Model, semantic model; Q-Model, quantitative model; CS-Model, clinical-semantic model; CQ-Model, clinical-quantitative model; SQ-Model, semantic-quantitative model; ACC, accuracy; SEN, sensitivity; SPE, specificity; PPV, positive predictive value; NPV, negative predictive value; AUROC, area under the receiver operating characteristic curve; AUPRC, area under the precision recall curve.

**Figure 5:**
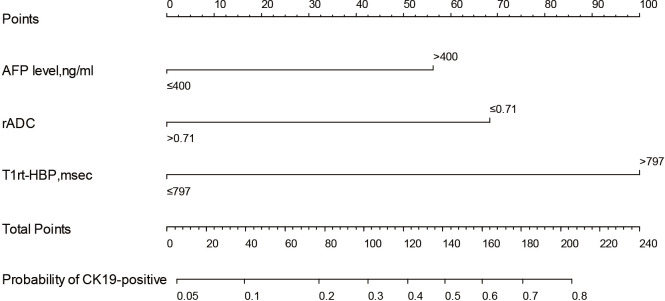
Nomogram of the CQ-Model. Predictor points are found on an uppermost point scale that corresponds to each variable. On the bottom scale, points for all variables are added and translated into the probability of CK19-positive HCC.

Based on CQ-Model, a nomogram was developed to predict CK19-positive HCCs (**Figure 5**), and the C-indexes in the training and test set were (0.844, 95%CI: 0.759-0.908) and (0.818, 95%CI: 0.693-0.902), respectively. The calibration curve showed that the probability of CK19-positive HCCs predicted by the CQ-Model was in good agreement with the actual probability (**Supplementary Figure S1**). Decision curve analysis demonstrated that CQ-Models provided larger net benefit across the range of reasonable threshold probabilities compared with the treat-all strategy and treat-none strategy (**Supplementary Figure S2**).

## 4 Discussion

In this study, we successfully developed and tested a nomogram based on T1 mapping on gadoxetic acid-enhanced MRI, which was used to individually predict CK19 expression in HCC, demonstrating good predictive efficiency and clinical utility. The nomogram is easy for clinical application and can facilitate personalized risk stratification and further treatment decisions for patients with CK19-positive HCC.

Our study showed that elevated preoperative serum AFP levels (>400ng/ml) were an independent factor of CK19-positive HCCs, which is consistent with previous studies (11). AFP is an important tumor marker for HCC and has been confirmed to be associated with CK19 expression. In HCC patients, elevated serum AFP level is positively correlated with poor differentiation, microvascular invasion and tumor recurrence (23, 24), which is consistent with the biological behavior of CK19-positive HCCs with high aggression. Through comparative analysis, we found that combining preoperative serum AFP level of HCC patients can improve the predictive performances of semantic and quantitative MRI model. In addition, we also found that nonsmooth tumor margin, target sign and peritumoral enhancement on MRI were closely correlated with CK19 status in HCC. Multivariable analysis based on semantic MRI features showed that target sign was an independent factor associated with CK19-positive HCCs, which was similar to the findings of Hu et al. (10). Previous studies have indicated that target sign was an important independent predictor for the diagnosis of intrahepatic cholangiocarcinoma (ICC), which is related to the pathological morphology of peripheral hyperproliferation and central stromal fibrosis (25, 26). The formation of stromal fibrosis seemed to be more common in CK19-positive HCCs than in CK19-negative HCCs, which indicated the morphological characteristics of CK19-positive HCC could be between those of typical HCC and ICC (5, 27). Based on semantic MRI features of HCC, the accuracy and sensitivity for prediction of CK19 expression were limited in our study, which was similar to the results of previous studies (9, 10); therefore, there remain limitations to its application in daily practice.

We conducted correlation analysis between quantitative MRI parameters and CK19 status, and the results showed that the correlation degree between quantitative parameters based on T1 mapping and CK19 status was higher than that based on signal intensity (SI). T1 relaxation time is an absolute value, which is not affected by scanning sequence parameters, and is proportional to the concentration of gadolinium contrast agent in the tissue (28), while SI is a relative value, the difference of technical factors will affect the value of SI, and does not have a linear relationship with the concentration of contrast agent, so T1 relaxation time is more accurate and reliable than SI. This has been demonstrated in the evaluation of liver function in patients with HCC and staging hepatic fibrosis (29, 30). In addition, the correlation between quantitative parameters based on T1 mapping and CK19 is also better than relative ADC values. The reason may be that T1 relaxation time reflects the inherent characteristics of tissues and can directly reflect the proliferation of tumor, while the ADC value can only indirectly reflect the proliferation of tumor through the diffusion of water molecules (31). therefore, the quantitative parameters based on T1 mapping are more closely related to the CK19 status. In general, T1 relaxation time of HCC correlated with CK19 status, and tumor proliferation was more active in CK19-positive HCCs compared with CK19-negative HCCs, resulting in prolonged T1 relaxation time. The univariable analysis of quantitative parameters showed that T1rt-HBP had the best efficiency for predicting CK19-positive HCCs, possibly because the T1 relaxation time was proportional to the concentration of gadolinium contrast agent, and more gadolinium contrast agent enters the tumor tissue at the HBP, thereby shortening the T1 relaxation time. Therefore, T1rt-HBP can more accurately predict CK19-positive HCCs.

To identified the best model for predicting CK19-positive HCCs, stepwise regressions based on the AIC were used to select variables to construct different predictive models. Our study showed that the CQ-model combining AFP and quantitative features had the best predictive performance. Some radiomics studies (13–16) showed that radiomics scores had good diagnostic efficacy in predicting CK19 expression. In the study by Wang W et al. (13), the combined model achieved a higher AUC than in our study for predicting CK19 expression (0.959 vs 0.844), albeit using a single-institution set. Besides, Yang F et al. (16). reported similar AUC for identifying CK19 status (0.857) with their radiomics signatures extracted from multisequence MRI, However, the AUCs of the two validation sets were only 0.726 and 0.790, respectively. Similarly, Chen Y et al. (19). achieved good results in predicting CK19 expression by DL model, but the DL model did not perform well in the test set (AUC), with AUCs of only 0.614 and 0.750 in the two test sets, respectively. The small sample size of the test set or the differences in MRI protocols may affect the robustness of the model. Undoubtedly, radiomics is a promising approach, but these studies are limited by image segmentation and standardization to the detriment of clinical practice. In contrast, our proposed CQ model is relatively easier to implement clinically, and the combination of quantitative MRI features (T1rt-HBP, rADC) and clinical information (AFP) can provide complementary information in building the model and improve the predictive performance. Meanwhile, the application of the CQ-Model in external test data demonstrated good robustness with different MRI scanners and with different parameter settings. We suggest that T1 mapping can be integrated as an additional protocol in gadoxetic acid-enhanced MR imaging for evaluating CK19 expression status in HCC, and T1 relaxation time is expected to provide additional information for predicting CK19-positive HCCs, which is preliminary and warrants further validation.

There were several limitations to our study. First, this is a retrospective study and only single HCC was selected, which may have selection bias in data homogeneity. Second, the small sample size of the test set may affect the robustness of model. Therefore, the prediction model needs to be further optimized through large-scale and multicenter studies in future studies. Third, we did not calculate the T1 relaxation time ratio of tumor to liver parenchyma because the presence of liver cirrhosis might have some influence on the results.

## 5 Conclusions

In summary, T1 mapping on gadoxetic acid-enhanced MRI combined with Diffusion-weighted Imaging and AFP can help predict the CK19 expression status in HCC, which is expected to provide important guiding value for the subsequent treatment and prognostic assessment of HCC patients. These results warrant further validation in future randomized trials to test the clinical utility of our imaging signature in combination with clinical-radiologic criteria to guide individualized therapeutic selection.

## Data availability statement

The original contributions presented in the study are included in the article/**Supplementary Material**. Further inquiries can be directed to the corresponding author.

## Ethics statement

The studies involving human participants were reviewed and approved by The Ethics Review Board of Central People’s Hospital of Zhanjiang. Written informed consent for participation was not required for this study in accordance with the national legislation and the institutional requirements.

## Author contributions

XT, JC, and YZ conducted the literature search. XT, JC, YZ, HH, PL and XJ designed the study. XT, JC, PL, and HH collected the data. XT, JC, YZ, HH, PL, and YL analyzed the data. All authors verified the data. XT, JC, YZ, PL, HH, and YL edited the manuscript. HT, YL, XT, and YZ reviewed the manuscript. All authors contributed to the article and approved the submitted version.
