# Supplementary material for: Preoperative prediction of cytokeratin-19 expression for hepatocellular carcinoma using T1 mapping on gadoxetic acid-enhanced MRI combined with diffusion-weighted imaging and clinical indicators
Source: Front Oncol. 2023 Jan 19;12:1068231. doi: 10.3389/fonc.2022.1068231 (PMC9893005; doi:10.3389/fonc.2022.1068231)
Supplement: Supplementary file 1 [file DataSheet_1.docx]

Supplementary Material

# Supplementary Methods

## MRI protocols of the two Institutions

All study patients from institutions I and II underwent gadoxetic acid-enhanced MRI using 3.0-T or 1.5-T systems. Technical details of MRI protocols at the two institutions are outlined in Table S1.

**TABLE S1** MR scan sequence and parameters

| Acquisition Sequence | Matrix Size | Section Thickness (mm) | Intersection Gap (mm) | Repetition Time  (msec) | Echo Time  (msec) | Flip Angle (º) |
| --- | --- | --- | --- | --- | --- | --- |
| Magneton Trio A Tim 3.0-T system (Siemens Healthcare, Erlangen, Germany) | | | | | | |
| T_1_WI-VIBE | 320×240 | 3 | 1 | 4.00 | 1.29/2.52 | 9 |
| T_1_WI-TWIST-VIBE | 288×216 | 3 | 0 | 3.89 | 1.23/ 2.46 | 13 |
| T_2_WI-HASTE | 320×240 | 3 | 1 | 3637 | 87 | 140 |
| DWI (b=50, 800 sec/mm^2^) | 128×128 | 5 | 1 | 6200 | 50 | / |
| T_1_ mapping (MOLLI) | 224×168 | 4 | 1 | 5.01 | 2.3 | (3, 15) |
| Magneton Aera 1.5-T system (Siemens Healthcare, Erlangen, Germany) | | | | | | |
| T_2_W1 | 320×224 | 5 | 1 | 2000 | 81 | 150 |
| GRE T_1_WI IN/OUT PHASE | 192×256 | 5 | 1 | 200 | 2.2/1.1 | 65 |
| DWI (b=50, 800 sec/mm^2^) | 192×154 | 5 | 1 | 5100 | 73 | / |
| T1WI-VIBE | 320×208 | 3 | 0 | 3.3 | 1.2 | 15 |
| T_1_ mapping (MOLLI) | 256×204 | 3 | 1 | 3.37 | 1.18 | (2, 11) |

Abbreviation: VIBE, volumetric interpolated breath-hold examination; TWIST, time-resolved imaging with interleaved stochastic trajectories; HASTE, half-Fourier acquisition single-shot turbo spin-echo; DWI, diffusion-weighted imaging; GRE, gradient recalled echo; MOLLI, modified look-locker inversion recovery.

## Image Analysis

All MRI images were independently reviewed by two board-certified radiologists (reader 1 [TAN XL] and reader 2 [CHEN JM] both with 6 years of experience in liver imaging, respectively) to evaluate the following characteristics for each HCC.

**The semantic MRI features included:**

1. **Tumor margin**: categorized as smooth margin and nonsmooth margin on hepatobiliary phase (HBP) images, round or oval tumors with smooth contour were identified as smooth margin (type Ⅰ), while nonsmooth tumor margins included local protruding type (type Ⅱ), polynodular fusion type (type Ⅲ) and irregular infiltrating type (type Ⅳ);
2. **Hemorrhage**: high signal intensity (SI) on T_1_-weighted imaging (T_1_WI) with variable SI on T_2_-weighted imaging (T_2_WI);
3. **Necrosis**: defined as high SI areas inside the lesion on T_2_WI or low SI areas not enhanced during enhancement;
4. **Fat component**: compared with the in-phase images, the tumor area had decreased SI on out-phase images;
5. **Target signs**: ring-like high SI with central relative hypointensity on diffusion-weighted imaging (DWI); Moderate to marked hypointensity in the periphery and mild hypointensity in the central area on HBP;
6. **Wash out**: The enhancement of lesions in the AP was reduced and lower than that in the peripheral liver parenchyma in the portal phase (PVP) or equilibrium phase (EP);
7. **Peritumoral enhancement**: irregular and patchy peritumoral hyperenhancement on the arterial phase (AP) that became isointense with normal liver parenchyma in the PVP or EP;
8. **Rim Arterial phase hyperenhancement (rim APHE)**: ring-like enhancement with central relative hypointensity;
9. **Intratumoral arteries**: visible intratumor blood vessel on the AP;
10. **Tumor capsule**: smooth, uniform, sharp border around most or all of the tumor, visible as an enhancing rim in the PVP or EP, and classified as capsular (complete or incomplete capsule) or non-capsular;
11. **Peritumoral hypointensity on HBP**: irregular or wedge-shaped hypointense areas around the tumor on HBP images;

**The quantitative MRI features included:**

1. Tumor size: maximum dimension measured on the coronal or axial plane on HBP images;
2. The SIs of tumor and normal liver parenchyma were measured on pre-enhancement, AP, PVP, EP and HBP images respectively, and the following quantitative parameters were calculated:

Tumor to liver contrast ratio (TLR): $\frac{Tumor\_SI \text{post}}{Liver\_SI\text{ }\text{post}}$

1. Tumor to liver apparent diffusion coefficient ratio (rADC): $\frac{Tumor\_ADC}{Liver\_ADC}$
2. Reduction rate of T_1_ relaxation time (rrT_1_rt):$\frac{(T\text{1}rt\_Pre - T\text{1}rt\_HBP)}{T\text{1}rt\_pre}$

# Supplementary Results

## Supplementary Tables

**TABLE S2** Univariate analyses of clinical characteristics in the training set.

| Variables | CK19-positive HCC (n = 35) | CK19-negative HCC (n = 67) | *p* value |
| --- | --- | --- | --- |
| Age (years) | 54 (47 ~ 65) | 55 (50 ~ 66) | 0.450 |
| Gender (male) | 31 (88.6%) | 62 (92.5%) | 0.503 |
| HBsAg |  |  | 0.923 |
| Negative | 6 (17.1%) | 12 (17.9%) |  |
| Positive | 29 (82.9%) | 55 (82.1%) |  |
| ALT (U/l) | 31.00 (20.50 ~ 55.75) | 37.00 (26.50 ~ 56.00) | 0.350 |
| AST (U/l) | 42.00 (31.00 ~ 48.25) | 38.00 (24.50 ~ 49.00) | 0.405 |
| GGT (U/l) | 49.00 (38.00 ~ 90.00) | 89.00 (31.50 ~ 139.00) | 0.223 |
| ALP (U/l) | 90.50 (71.00 ~ 113.50) | 80.50 (63.75 ~ 98.50) | 0.091 |
| ALB (g/l) | 39.40 (36.00 ~ 42.60) | 39.80 (36.30 ~ 42.42) | 0.521 |
| TBIL (umol/l) | 14.90 (11.69 ~ 17.70) | 14.62 (11.32 ~ 18.12) | 0.685 |
| DBIL (umol/l) | 4.36 (2.40 ~ 7.98) | 4.64 (2.90 ~ 6.75) | 0.547 |
| SCr (U/l) | 75.00 (64.00 ~ 87.12) | 75.50 (67.15 ~ 86.90) | 0.592 |
| PT (s) | 11.80 (11.50 ~ 12.60) | 12.00 (11.30 ~ 12.40) | 0.738 |
| INR |  |  | 0.861 |
| ≤ 1.0 | 14 (40.0%) | 28 (41.8%) |  |
| ＞1.0 | 21 (60.0%) | 39 (58.2%) |  |
| NLR | 2.49 (1.88 ~ 3.61) | 1.88 (1.29 ~ 2.70) | 0.009* |
| PLR | 123.49 (78.06 ~ 178.62) | 104.14 (70.85 ~ 141.38) | 0.138 |
| AFP (ng/ml) | 120.10 (60.70 ~ 1706.41) | 14.50 (4.32 ~ 42.93) | 0.007* |

Notes: **p*<0.05. Continuous variables are presented as median (inter-quartile range, IQR). Categorial variables are presented as number (percentage).

Abbreviations: HBsAg, hepatitis B surface antigen; ALT, alanine aminotransferase; AST, aspartate aminotransferase; GGT, glutamyl transpeptidase; ALP, alkaline phosphatase; ALB, albumin; TBIL, total bilirubin; DBIL, direct bilirubin; SCr, serum creatinine; PT, prothrombin time; INR, international normalized ratio; NLR, neutrophil to lymphocyte ratio; PLR, platelet to Lymphocyte ratio; AFP, alpha fetoprotein; CK19, cytokeratin 19.

**TABLE S****3** Comparison of semantic MRI findings between CK19-negative and positive HCCs in training set.

| Features | CK-19 negative  (n = 67) | CK-19 positive  (n = 35) | *p* value |
| --- | --- | --- | --- |
|  |  |  |  |
| Nonsmooth tumor margin | 42 (62.7%) | 29 (82.9%) | 0.035* |
| Hemorrhage | 21 (31.3%) | 9 (25.7%) | 0.544 |
| Necrosis | 26 (38.8%) | 19 (54.3%) | 0.135 |
| Fat component | 13 (19.4%) | 6 (17.1%) | 0.781 |
| Target sign | 13 (19.4%) | 16 (45.7%) | 0.005* |
| Rim APHE | 31 (46.3%) | 23 (65.7%) | 0.062 |
| Corona enhancement | 28 (41.8%) | 22 (62.9%) | 0.043* |
| Intratumoral arteries | 36 (53.7%) | 22 (62.9%) | 0.377 |
| Washout | 51 (76.1%) | 23 (65.7%) | 0.264 |
| Radiologic capsule | 56 (83.6%) | 26 (74.3%) | 0.262 |
| Peritumoral hypointensity on HBP | 24 (35.8%) | 17 (48.6%) | 0.212 |

Note: **p*<0.05. Except where indicated, data are numbers of patients, with percentages in parentheses.

Abbreviations: HCC, hepatocellular carcinoma; CK19, cytokeratin 19; APHE, arterial phase hyperenhancement; HBP, hepatobiliary phase.

**TABLE S4** Correlation between quantitative MRI parameters and CK19-positive expression.

|  | CK19-positive expression (n = 102) | | |
| --- | --- | --- | --- |
|  | r | 95% CI | *p* value |
| Tumor size (cm) | 0.121 | -0.081 ~ 0.314 | 0.224 |
| rADC | -0.358 | -0.476 ~ -0.227 | <0.001*** |
| AP-TLR | -0.112 | -0.288 ~ 0.085 | 0.265 |
| PVP-TLR | -0.171 | -0.349 ~ 0.014 | 0.088 |
| EP-TLR | -0.128 | -0.313 ~ 0.055 | 0.200 |
| HBP-TLR | -0.309 | -0.494 ~ 0.129 | 0.002** |
| T1rt-Pre | 0.352 | 0.167 ~ 0.518 | <0.001*** |
| T1rt-HBP | 0.366 | 0.192 ~ 0.561 | <0.001*** |
| rrT1rt | -0.065 | -0.266 ~ 0.121 | 0.519 |

Notes: *** At 0.001 level (two-tailed), the correlation was significant.

** At 0.01 level (two-tailed), the correlation was significant.

* At 0.05 level (two-tailed), the correlation was significant.

Abbreviations: CK-19, cytokeratin 19; ADC, apparent diffusion coefficient; rADC, relative ADC; AP, arterial phase; PVP, portal venous phase; EP, equilibrium phase; HBP, hepatobiliary phase; TLR, tumor to liver contrast ratio; TEI, tumor enhancement index; RTE, relative tumor enhancement; RER, relative enhancement ratio; Pre, pre-enhancement; T1rt, T1 relaxation time; rrT1rt, reduction rate of T1rt.

**TABLE S5** Diagnostic of significant quantitative MRI parameters for predicting CK19-positive HCC in the training set.

|  | AUROC (95% CI) | SEN | SPE | Youden | cut-off | *p* value |
| --- | --- | --- | --- | --- | --- | --- |
| rADC | 0.710 (0.610 ~ 0.794) | 68.57 | 68.66 | 0.37 | ≤0.71 | ＜0.001 |
| HBP-TLR | 0.681 (0.582 ~ 0.770) | 74.29 | 59.70 | 0.34 | ≤0.58 | 0.002 |
| T1rt-Pre | 0.709 (0.611 ~ 0.795) | 62.86 | 74.63 | 0.37 | ＞1437 msec | ＜0.001 |
| T1rt-HBP | 0.712 (0.614 ~ 0.797) | 80.00 | 62.69 | 0.43 | ＞797 msec | ＜0.001 |

Abbreviations: *HCC*, hepatocellular carcinoma; *CK19*, cytokeratin 19; *AUROC*, area under the receiver operating characteristic curve; *CI,* confidence interval; *SEN*, sensitivity; *SPE*, specificity; *ADC*, apparent diffusion coefficient; *rADC*, relative ADC; *HBP*, hepatobiliary phase; *TLR*, tumor to liver contrast ratio; *TEI*, tumor enhancement index; *RTE*, relative tumor enhancement; *RER*, relative enhancement ratio; *Pre*, pre-enhancement; *T1rt*, T1 relaxation time.

**TABLE S6** Multivariable logistic regression analysis for predicting CK19-positive HCCs in training set.

|  | β value | SE | *p* value | OR（95% CI） |
| --- | --- | --- | --- | --- |
| AFP＞400ng/ml | 1.527 | 0.732 | 0.037* | 4.607 (1.098 ~ 19.326) |
| NLR＞1.86 | 0.533 | 0.644 | 0.408 | 1.703 (0.482 ~ 6.016) |
| Nonsmooth tumor margin | 0.743 | 0.727 | 0.307 | 2.102 (0.505 ~ 8.743) |
| Corona enhancement | -1.020 | 0.695 | 0.142 | 0.361 (0.092 ~ 1.407) |
| Target sign | 0.700 | 0.642 | 0.275 | 2.015 (0.573 ~ 7.089) |
| HBP-TLR≤0.58 | 0.697 | 0.582 | 0.232 | 2.007 (0.641 ~ 6.282) |
| rADC≤0.71 | 1.238 | 0.571 | 0.030* | 3.450 (1.126 ~ 10.567) |
| T1rt-Pre＞1437 msec | 1.090 | 0.578 | 0.059 | 2.974 (0.957 ~ 9.235) |
| T1rt-HBP＞797 msec | 1.506 | 0.634 | 0.018* | 4.509 (1.301 ~ 15.626) |

Notes: **p*<0.05.

Abbreviations: HCC, hepatocellular carcinoma; CK19, cytokeratin 19; AFP, alpha fetoprotein; NLR, neutrophil to lymphocyte ratio; HBP, hepatobiliary phase; TLR, tumor to liver contrast ratio; rADC, relative apparent diffusion coefficient; T1rt, T1 relaxation time; Pre, pre-enhancement; SE, standard Error; OR, odd ratio; CI, confidence interval.

**TABLE S7** Selection process of CK19-positive HCCs prediction model in the training set.

| Model | Variable | AIC |
| --- | --- | --- |
| C-Model | AFP+NLR | 117.04 |
| S-Model | nonsmooth_tumor_margin + target_sign + corona_enhancement | 126.86 |
|  | nonsmooth_tumor_margin + target_sign | 126.14* |
| Q-Model | T1rt_Pre + T1rt_HBP + HBP_TLR + rADC | 107.43 |
|  | T1rt_Pre + T1rt_HBP + rADC | 106.83* |
| CS-Model | AFP + NLR + nonsmooth_tumor_margin + target_sign + corona_enhancement | 118.89 |
|  | AFP + NLR + nonsmooth_tumor_margin + target_sign | 116.91 |
|  | AFP + NLR + nonsmooth_tumor_margin | 115.55* |
| CQ-Model | AFP + NLR + T1rt_Pre + T1rt_HBP + HBP_TLR + rADC | 103.88 |
|  | AFP + T1rt_Pre + T1rt_HBP + HBP_TLR + rADC | 102.63 |
|  | AFP + T1rt_HBP + HBP_TLR + rADC | 102.02 |
|  | AFP + T1rt_HBP + rADC | 101.75* |
| SQ-Model | nonsmooth_tumor_margin + target_sign + corona_enhancement + T1rt_Pre + T1rt_HBP + HBP_TLR + rADC | 107.64 |
|  | nonsmooth_tumor_margin + target_sign + T1rt_Pre + T1rt_HBP + HBP_TLR + rADC | 106.92 |
|  | nonsmooth_tumor_margin + target_sign + T1rt_Pre + T1rt_HBP + rADC | 106.19 |
|  | nonsmooth_tumor_margin + T1rt_Pre + T1rt_HBP + rADC | 105.70* |
| CSQ-Model | AFP + NLR + nonsmooth_tumor_margin + target_sign + corona_enhancement + T1rt_Pre + T1rt_HBP + HBP_TLR + rADC | 105.86 |
|  | AFP + nonsmooth_tumor_margin + target_sign + corona_enhancement + T1rt_Pre + T1rt_HBP + HBP_TLR + rADC | 104.55 |
|  | AFP + target_sign + corona_enhancement + T1rt_Pre + T1rt_HBP + HBP_TLR + rADC | 103.65 |
|  | AFP + corona_enhancement + T1rt_Pre + T1rt_HBP + HBP_TLR +rADC | 103.38 |
|  | AFP + T1rt_Pre + T1rt_HBP + HBP_TLR + rADC | 102.63 |
|  | AFP + T1rt_Pre + T1rt_HBP + rADC | 102.02 |
|  | AFP + T1rt_HBP + rADC | 101.75* |

Notes: *The minimum AIC value for each model.

Abbreviations: HCC, hepatocellular carcinoma; CK19, cytokeratin 19; AIC, Akaike information criterion; C-Model, Clinical model; S-Model, semantic model; Q-Model, quantitative model; CS-Model, clinical-semantic model; CQ-Model, clinical-quantitative model; SQ-Model, semantic-quantitative model; CSQ-Model, semantic-semantic-quantitative model.

**TABLE S8** Comparison of the performance of prediction models based on ROC curves by DeLong's test (*p*-values presented).

| Models |  | C-Model | S-Model | Q-Model | CS-Model | CQ-Model | SQ-Model |
| --- | --- | --- | --- | --- | --- | --- | --- |
|  | AUROC | 0.718 | 0.715 | 0.814 | 0.772 | 0.845 | 0.826 |
| C-Model | 0.718 |  | 0.304 | 0.282 | 0.103 | 0.036* | 0.177 |
| S-Model | 0.715 |  |  | 0.067 | 0.084 | 0.016* | 0.026* |
| Q-Model | 0.814 |  |  |  | 0.502 | 0.307 | 0.271 |
| CS-Model | 0.772 |  |  |  |  | 0.136 | 0.346 |
| CQ-Model | 0.845 |  |  |  |  |  | 0.505 |
| SQ-Model | 0.826 |  |  |  |  |  |  |

Notes: **p*<0.05. Values in table indicate the significance level of the AUROCs comparison between different two models.

Abbreviations: *AUROC*, area under the receiver operating characteristic curve; *C-Model,* Clinical model; *S-Model*, semantic model; *Q-Model*, quantitative model; *CS-Model*, clinical-semantic model; *CQ-Model*, clinical-quantitative model; *SQ-Model*, semantic-quantitative model.

## Supplementary Figures


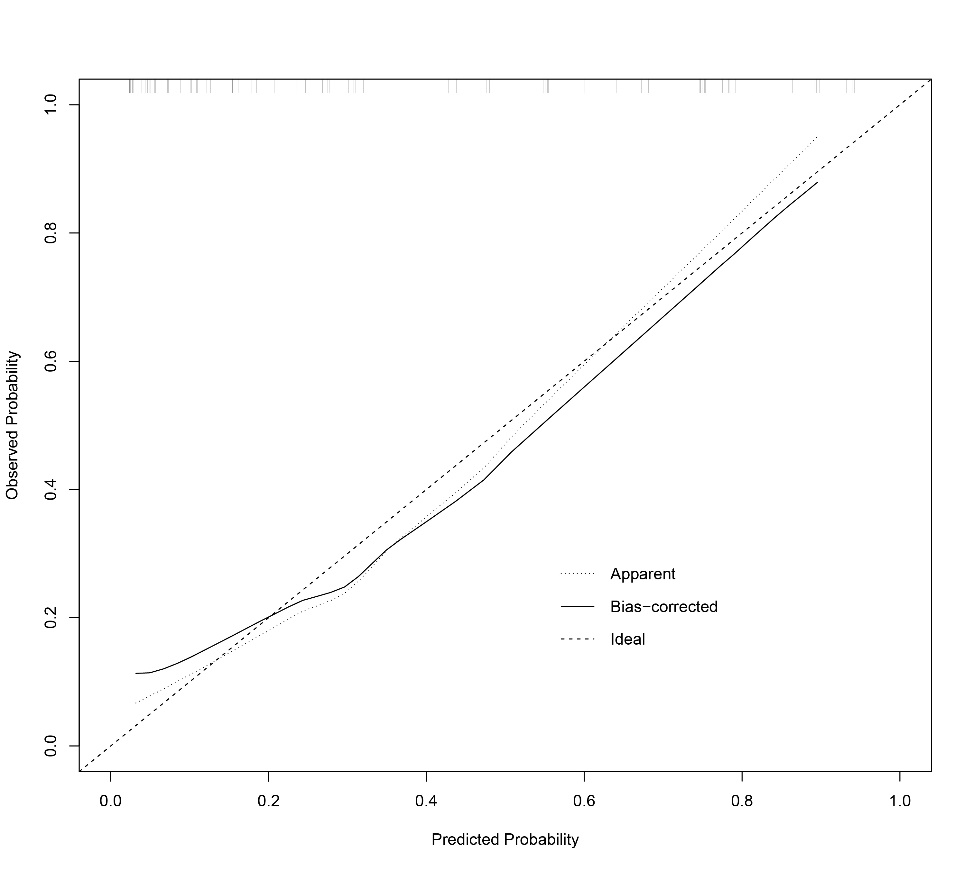


**A**


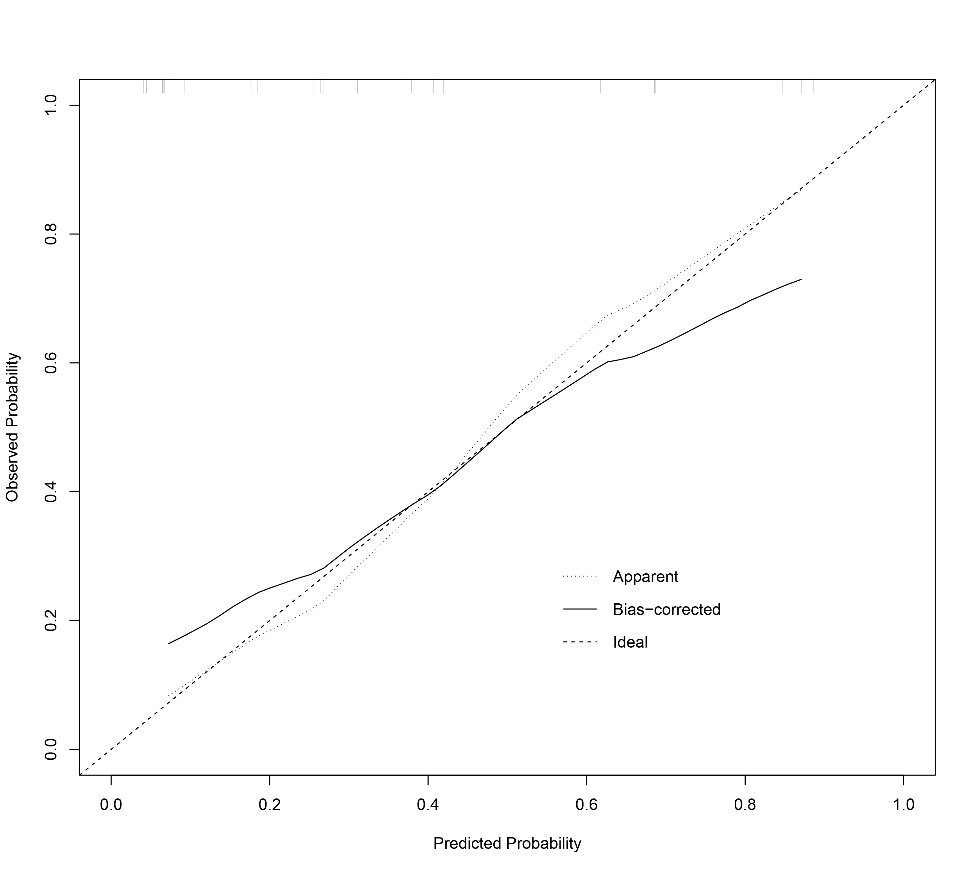


**B**

**Supplementary Figure S1.** Calibration curves of the nomogram based on CQ-Model in the training (**A**) and test (**B**) sets. The y-axis shows the actual result. The x-axis represents the predicted probability. The diagonal dotted line represents the reference line showing the “ideal” prediction. The solid line indicates the performance of the nomogram. If the solid line is closer to the diagonal dotted line, it means a better prediction.


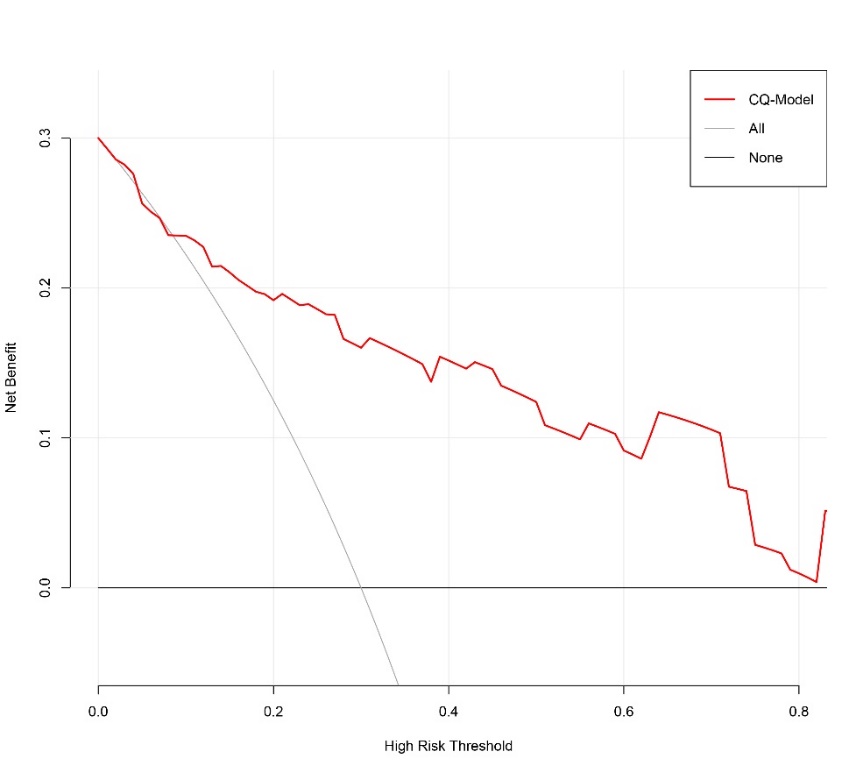


**A**


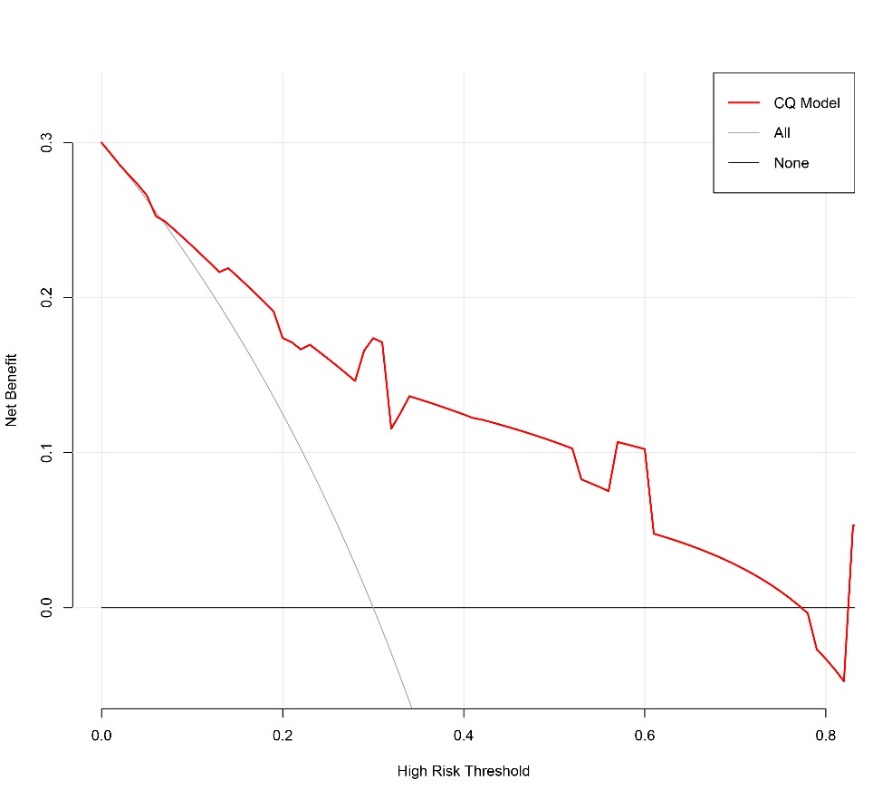


**B**

**Supplementary Figure S2.** Decision curve analysis of clinical usefulness assessment of the nomogram based on CQ-Model in the training (**A**) and test (**B**) sets. The y-axis represents the net benefit, and the x-axis represents the threshold probability. The nomogram based on CQ-Model achieves more net benefit across the majority of the range of threshold probabilities compared with the treat-all strategy and treat-none strategy.
